# Signature Inspired Home Environments Monitoring System Using IR-UWB Technology

**DOI:** 10.3390/s19020385

**Published:** 2019-01-18

**Authors:** Soumya Prakash Rana, Maitreyee Dey, Mohammad Ghavami, Sandra Dudley

**Affiliations:** Biomedical Engineering and Communications (BiMEC) Research Centre, School of Engineering, London South Bank University, 103 Borough Road, London SE1 0AA, UK; deym@lsbu.ac.uk (M.D.); ghavamim@lsbu.ac.uk (M.G.); dudleyms@lsbu.ac.uk (S.D.)

**Keywords:** Edge Care System, Ultra-Wide Band, Indoor Location, Movement Detection, Support Vector Machine

## Abstract

Home monitoring and remote care systems aim to ultimately provide independent living care scenarios through non-intrusive, privacy-protecting means. Their main aim is to provide care through appreciating normal habits, remotely recognizing changes and acting upon those changes either through informing the person themselves, care providers, family members, medical practitioners, or emergency services, depending on need. Care giving can be required at any age, encompassing young to the globally growing aging population. A non-wearable and unobtrusive architecture has been developed and tested here to provide a fruitful health and wellbeing-monitoring framework without interfering in a user’s regular daily habits and maintaining privacy. This work focuses on tracking locations in an unobtrusive way, recognizing daily activities, which are part of maintaining a healthy/regular lifestyle. This study shows an intelligent and locally based edge care system (ECS) solution to identify the location of an occupant’s movement from daily activities using impulse radio-ultra wide band (IR-UWB) radar. A new method is proposed calculating the azimuth angle of a movement from the received pulse and employing radar principles to determine the range of that movement. Moreover, short-term fourier transform (STFT) has been performed to determine the frequency distribution of the occupant’s action. Therefore, STFT, azimuth angle, and range calculation together provide the information to understand how occupants engage with their environment. An experiment has been carried out for an occupant at different times of the day during daily household activities and recorded with time and room position. Subsequently, these time-frequency outcomes, along with the range and azimuth information, have been employed to train a support vector machine (SVM) learning algorithm for recognizing indoor locations when the person is moving around the house, where little or no movement indicates the occurrence of abnormalities. The implemented framework is connected with a cloud server architecture, which enables to act against any abnormality remotely. The proposed methodology shows very promising results through statistical validation and achieved over 90% testing accuracy in a real-time scenario.

## 1. Background

Developments in life expectancy, disability awareness, and the support for independent living has altered how users, who may require care, are provided that care. Independent living with remote care provision and support are highly sought goals in caring for those who need it today and in the future. Background support enables those in need to go about their daily life knowing that help is there if required, giving people the confidence to go about their normal daily lives. Ambient assisted living (AAL) is a significant area of research and development aiming to apply ambient intelligence technology, enabling those with varying care needs, such as older persons, to live in their preferred environment for longer and safely [1,2]. ECS systems can use different types of sensors to monitor the movement and daily health aspects of users requiring remote care. These sensors can be classified into two groups; (1) sensors, which are at fixed at a particular location, e.g., passive infrared (PIR) sensors, vibration sensors, pressure sensors, cameras, and microphones; and (2) mobile and wearable sensors, e.g., accelerometers, thermal sensors, and pulse oximeters. Sensing based investigations for example, received signal strength indicator (RSSI) to localize people [3,4], acoustic sensor to localize animals [5], and adaptive rejection sampling (ARS) for target navigation are gaining popularity [6].

There are several sensors or sensor combinations available. Currently there are plentiful ECS systems implementing various tasks, such as fall detection [7,8], mobile emergency response [9], video surveillance [10], automation [11], monitoring activities of daily living [12], and respiratory monitoring [13]. These attempts can increase the safety and independence for the elderly life. In addition, there are several protocols existing to deliver older care services, which uses multiple sensors with machine learning algorithms to get health status of a person and some of these systems could be operated remotely. Tsirmpas et al. created an AAL system to classify various activities from the data generated using accelerometer. They have made profiles of activities from accelerometer data and classified the profiles using self-organizing map (SOM) and fuzzy C-means (FCM) algorithms [14]. Costa et al. constructed a care system to detect falls and health condition using multiple wearable devices for old people which supports mobility. The system uses a chest band, a smart shoe, and an accelerometer enabled smart phone simultaneously to obtain bio-signals and generates notification for the occurrence of any abnormalities [15]. Yao et al. modeled another fall detection method in the context of AAL with the help of Kinect depth sensor (D sensor) and machine learning. The RGB video was collected for humans by enabling D sensor which provides the skeleton data (coordinates of joints) and classified using interval type-2 fuzzy-logic-based systems (IT2FLSs) to track the behaviors of people in home [16]. Diamantini et al. employed a formal language approach to form a requirements elicitation system for AAL and an ontology of elderly people’s daily behavior. The system divides the tasks and implements logical reasoning to support the ECS [17]. Alcalá et al. and Lopez-de-Teruel et al. created a non-intrusive ECS employing a smart meter and artificial intelligence. The model gathers the power consumption from houses of healthy elderly residents and analyzed appliance power usage probabilistically with the help of Gaussian mixture model and the Dempster-Shafer theory. This system creates notifications to check on a person’s condition when power consumption deviates from usual usage, because this deviation may indicates a change in a normal routine [18,19]. Bleda et al. proposed an ECS by using smart sensory furniture (SSF). The experiment conducted in an elderly care home where the sensors are embedded with furniture to explore the interaction of people with their furniture and make a protocol for providing safety, prevention, and elderly care services. Specifically, this work added a middleware in their previously built infrastructure to provide an elder care facility [20]. Hassan et al. assembled a cloud based hybrid approach to take care of elderly people. The model used several ambient sensors including CCTV videos together to analyze patient’s condition. Then, the data was classified using Weka machine learning tools to take decisions about health status and generate alerts for any abnormal pattern found from the house [21]. Barsocchi et al. presented models where abnormal situations were detected through swarm intelligence and a marker based indoor navigation system [22] by implementing three models CPS [23], n-Core [24], and RealTrac [25] evaluating AAL Systems through Competitive Benchmarking (EvAAL). Diraco et al. created a prototype to monitor the health condition of older people using IR-UWB phenomenon when they were alone in their home. This work focused on AAL by measuring vital signs (heart rate and respiration rate) and fall detection. Subsequently, the data obtained from the UWB device were classified by supervised and unsupervised machine learning algorithms to identify unexpected and potentially dangerous situations [26]. Chernbumroong et al. published work on an experiment to detect of Activities of Daily Livings (ADLs) of an older person via wearable, inexpensive, and non-intrusive wrist worn sensors. The data were classified by multi-layer perceptron (MLP), radial basis function (RBF), and SVM to classify the activities to aid understanding of unusual conditions [27]. Fleury et al. performed experiments in health smart homes to categorize ADLs using SVM. Different classes, such as sleeping, toilet use, hygiene, resting, communication, eating, and dressing/undressing), were considered for the test [28].

### 1.1. Scope

Generally, care systems require context aware information, e.g., indoor location, activities, and contact timings of a person with furniture or other object to understand the lifestyle of users through machine learning or manual processing. Most of the existing care systems use wearable technologies to obtain context-aware information from the home environment. However, wearable devices are nowadays criticized for their low battery life and user dissatisfaction. Moreover, the devices face problems such as, coverage area, bandwidth, and integration with existing infrastructure. Smart phone sensors (e.g., accelerometer, received signal strength indicator (RSSI)) face a crucial disadvantage of the recalculation of signal strength at the time of environment changes, where cellular devices are not reliable because of altering signal propagation in different conditions and the fact that they might be left behind by the user in a single room when not in use. The systems based on non-wearable devices e.g., smart meters, smart furniture, and video tracking also suffer from the problems such as, cost of installation, maintenance and for example with smart meters, information only available every 30 min or so.

### 1.2. Contribution

The health care domain requires technologies which are acceptable to the user, cost effective in terms of overhead and data, and easily maintainable. The proposed work has chosen UWB as a fruitful and powerful method to accommodate drawbacks of the existing algorithms. The UWB radar used for the proposed work, functions as a non-intrusive biosensor detecting physiological movement in a noisy or multipath environment. The experimental setup has been made in a real home environment, which is connected via an Internet of things (IoT) platform, and brings much greater intelligence and understanding to identify a person’s condition (static or dynamic) over time and provides an assistance route via remote access control when needed. The work is an extended version of [29,30], where the initial work focused on to an automated UWB localization framework based on supervised machine learning and the second aimed to recognize vital signs (respiration and heart rate) during different daily activity types via UWB radar response. The proposed work here has extended those previous works above to an ECS improving AAL by developing trigonometric approach in accordance with radar principles and machine learning. This paper presents a new intelligent ECS mechanism via device-free passive (DfP) indoor localization [31] method where persons do not need to carry any devices nor join-in centralized infrastructure. In addition, it is robust to changes in the environment, does not need frequent manual care or reconstruction, which reduces huge overhead. The main contributions of this work are as follows:

- A pilot study has been performed in a real home environment with the presence of a person. Data have been collected for different types of activities via UWB radar and video surveillance (to ensure correlation of finding) to understand the "habitual" position through the daily activities.
- Radar principle has been employed to measure the range, and a new method has been proposed to calculate the azimuth angle or angle of arrival (AoA) from the pulse propagation delay in accordance with the time-stamp to identify the locations. Consequently, the experiment can explore the actual position of the person in different times, which would imply a normal movement.
- Subsequently, the raw data have been processed using short term fourier transform (STFT) to understand the frequency signature of an action. The frequency distribution of an activity along with the range, azimuth, and time-stamp of the movement have been labelled by the recorded evidence and made the ground-truth information.
- Subsequently, a multi class support vector machine (MC-SVM) has been trained and tested including the time-stamp of the daily "habitual" positions in that indoor scenario to make the system automated.
- The proposed method has been validated via statistical metrics and is shown to achieve over 90% accuracy.

The remainder of the paper is organized as follows. Section 2 highlights the methodology proposed and provides details regarding the time-frequency analysis along with the classification algorithm. Section 3 discusses the experimental set-up and detailed data acquisition process. In Section 4 the results obtained through frequency signature, classification, and validation process are presented. Section 5 concludes the paper and provides the future research directions of this work.

## 2. Proposed Work

This section describes the UWB radar functionality and its transformation, which are closely connected; hence, they are better understood by discussing them jointly. STFT is used to characterize and manipulate the local section of radar scans whose statistics vary in time. Once the frequency contents are determined by the STFT, the range and azimuth are calculated with the help of general radar principles and trigonometric comprehension of the user space. Then, the extracted information is fed into the SVM algorithm for automation purposes. A brief description is presented in the following sections.

### 2.1. Short-Time Fourier Transform (STFT)

STFT is a respected time frequency analysis tool [32]. In the present work, it generates important and distinct types of time-frequency distribution for different locations. The mathematical explanation of STFT is discussed below [33],

(1) $$S\left(a,f\right)=\sum_{n=-\infty }^{\infty }s\left(n\right)u\left(n-a\right)e^{-j2\pi fn}$$

where S(a,f)= frequency function, f= continuous variable denoting frequency, u(n−a)= window function, s(n)u(n−a)= short time section of s(n) at time *a*. Here, s(n) is the obtained from a room with a person presence sampled at *f* frequency with a particular interval. Subsequently, the shifted frequency or window (here a hamming window) is convoluted with the short term section of the signal to observe the frequency changes within a short term. Subsequently, the change of power in decibel (dB) has been determined using $20\times log_{10}\left(s\left(n\right)\right)$ for pulses where detection occurred to observe the change of power with respect to frequency for a human action.

### 2.2. Range and Azimuth Angle

The range [34] of the target, *R*, is determined by the round trip time of the received waveform. Therefore, the range of the moving objects are evaluated using $R=\frac{c△T}{2}$ by measuring the time delay where, $c=2.9\times 10^{8}$ m/s is the velocity of light, and △T is the time delay in seconds. Moreover, the angle of the moving object with the radar vision (azimuth) is determined using a trigonometric function and the radial plane.

**Figure 1:**
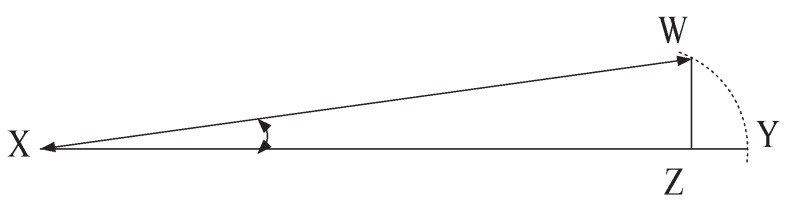
The geometry of azimuth angle.

Figure 1 displays the azimuth angle calculation to determine the position or orientation of moving body parts towards the radar. The spherical system measures the azimuth angle clockwise direction from the exact north of the receiver and is denoted by ϕ. The moving body part is deviated at ϕ, where the travelled distances are XY and XW in propagation delay $t_{1}$, $t_{2}$. Therefore, the change of the distance is (XY−XW)=YZ at the change of the time $(t_{1}-t_{2})=∆t$. The object is deviated from the exact north of the receiver. Now, YZ is approximately equivalent to the arc YW is created by the object at angle ϕ. Therefore, ϕ is calculated from the radian measure, and equivalent degree conversion is denoted in (2),

(2) $$\phi =\frac{YZ\times 360{}^{\circ}}{XY\times 2\pi }$$

### 2.3. Crammer and Singer’s MC-SVM

Here, the UWB localization data is considered as a multi-class categorization case. Therefore, the extracted features are fed into a Crammer and Singers MC-SVM, where a set of labelled training pattern is represented by $\left(x_{1},y_{1}\right),\cdots ,\left(x_{l},y_{l}\right)$ of cardinality *l*, where $x_{i}\in R^{d}$ and $y_{i}\in \left\{1,\cdots ,k\right\}$, $w\in R^{d}$ is the weight vector, $C\in R_{+}$ is the regularization constant, and φ is mapping function which projects training pattern into a suitable feature space *H* that allows for nonlinear decision surfaces. Crammer and Singer [35,36] proposed a SVM with multi categorization ability by solving the quadratic optimization problem as follows:

(3) $$\begin{array}{c} \min_{w_{m}\in H,\xi \in R^{l}}\;\;\;\frac{1}{2}\sum_{m=1}^{k}w_{m}^{T}w_{m}+C\sum_{i=1}^{l}\xi_{i}\;\\ \mathrm{subject}\;\mathrm{to}\;\;\;w_{y_{i}}^{T}\varphi \left(x_{i}\right)-w_{t}^{T}\varphi \left(x_{i}\right)\geq 1-\delta_{y_{i},t}-\xi_{i}\;\\ i=1,\cdots ,l\;\;\;\;\;;\;\;\;\;\;t\in 1,\ldots ,k\; \end{array}$$

where, $\{\delta_{i,j}$, j} is the Kronecker delta, defined as 1 for i=j and as 0 otherwise. The resulting decision function is defined as

(4) $${\mathrm{argmax}}_{m}f_{m}\left(x\right)={\mathrm{argmax}}_{m}w_{m}^{T}\varphi \left(x\right).$$

Note that the constraints $\xi_{i}\geq 0,i=1,\cdots ,l$, are implicitly indicated in the margin constraints of (3) when *t* equals $y_{i}$. Additionally, (3) focuses on classification rule (4) without any bias terms. A nonzero bias term may be readily modelled using an additional constant feature to each *x*. Thus, varying data categories are classified by solving this decision function with results analysed in the following section.

### 2.4. Performance Metrics

Performance rates of the proposed method has been statistically analyzed. Well-established statistical metrics are used to evaluate the proposed localization algorithm: Accuracy, sensitivity, specificity, positive predictive value (PPV), negative predictive value (NPV), and computation time all have been measured [37]. Sensitivity and specificity describe the ability of the proposed work to precisely recognize the room locations with activities at a given time. PPV and NPV signify the probability for correct identification by the system. The average of these metrics is considered in the Section 4 to justify the performance of the proposed work.

**Figure 2:**
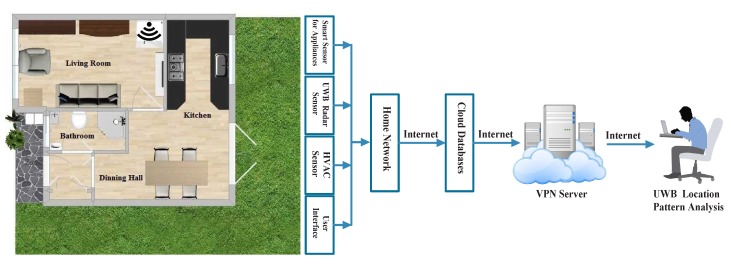
The secure cloud server and minimal internet of things (IoT) architecture refers to the components of the system embedded within the house comprising a ultra wide band (UWB) data collection front end, storage, and the post processing stages to understand home environment.

A pseudo code has been included in Algorithm 1 to discuss the generalization of the proposed prototype. The ranging and communications module (RCM) and the monostatic radar module (MRM) have been configured to start taking data using the settings of Table 1. This RCM and MRM module is connected with the network as shown in Figure 2. The module employs a graphical user interface (GUI) to collect the data. Then, an occupant person has performed a number of normal household tasks and the scan data have been gathered through the radar GUI.

The observation time has been noted along with the locations (living room, kitchen, etc.) during that period via simultaneous video for reference. The data file of each scan has been labelled by the noted information for further transformation and classification. The range and azimuth have been determined using (Algorithm 1, line numbers 11 and 12). The module follows two way time-of-flight (TW-TOF) mechanism and a number of data points, where the first 5 nanoseconds contain jitter due to the direct path interference between the transmitter and receiver antennas. Thus, data points prior to 5 nanoseconds have been filtered out from each radar scan during STFT conversion (Algorithm 1, line number 13). The range, azimuth angle, and frequency values have been then used as final features (Algorithm 1 line number 18). Next, the data have been randomly partitioned for training and testing. The training dataset has been employed to train the MC-SVM classifier (Algorithm 1, line number 21), and testing dataset to predict the location with activity. The outcomes have been validated (Algorithm 1, line number 24) by statistical measures. Subsequent to the training of MC-SVM and once a satisfied performance achieved, only the first and third phases are iterated automatically for location and activity prediction from real life data.

**Algorithm 1** Pseudo code of proposed method
**Require:** 
Configureradarmodule(usingTable1)
**Require:** 
ScandatafromRCM&MRMmodule
**Require:** 
Locationinfoofdatacollection

1:
**First Phase:**
2:
Totalnumberofscans=NoS
3:
$Number\;of\;datapoints\;per\;scan=D_{PS}$
4:
Rangeofdetection=R
5:
$Previously\;measured\;range,\;R_{p}=0$
6:
AzimuthorAoAofdetection=ϕ
7:
Propagationdelayindetection=∆T
8:
Numberofdatapointswithinfirst5ns=p
9:
Makegroundtruthofscandata
10:**for all**scan=1 **to** NoS**do**11: CalculateR(describedinSection4.2)12: Calculateϕ(usingEq.2)13: **for all** datapoints=(1+p) **to** $D_{PS}$ **do**14:  TransformdatapointsbySTFT(usingEq.1)15: **end for**16: $R_{p}=R$17:
**end for**
18:**Return,** Frequencydistributions,rangesR,azimuthsϕ≡Labelledfeatures19:
**Second Phase:**
20:
Maketraining&testingdataset
21:
TrainMC−SVM(usingEq.3&4)
22:
**Third Phase:**
23:
TestMC−SVMmodelbytestingdataset
24:
Validateresultsbymetrics(describedinSection4.4)



## 3. Experimental Setup

The previously outlined experiment has been carried out on the ground floor area of a semi-detached house located in Essex, UK, where the house is connected with several open source IoT devices such as smart and legacy appliances, sensor nodes, UWB platforms, user interface, and smart thermostat devices, etc. based on previous work presented [38]. Here, only the UWB platform is considered for this work. The ground floor plan, shown in Figure 2, comprises four rooms: Living room, kitchen, dining room, and a bathroom. The single monostatic UWB device is fixed towards the back corner of the living room. The data is accumulated with the presence and absence of a single person where the remainder of the environment is assumed static. The data are then collected and stored into a cloud database through middleware server architecture [39]. Later, the data are pre-processed, analysed, and transformed by a STFT and used to train the MC-SVM about the location information of the ground floor. Hence, the trained prototype could predict location of the future activities.

**Figure 3:**
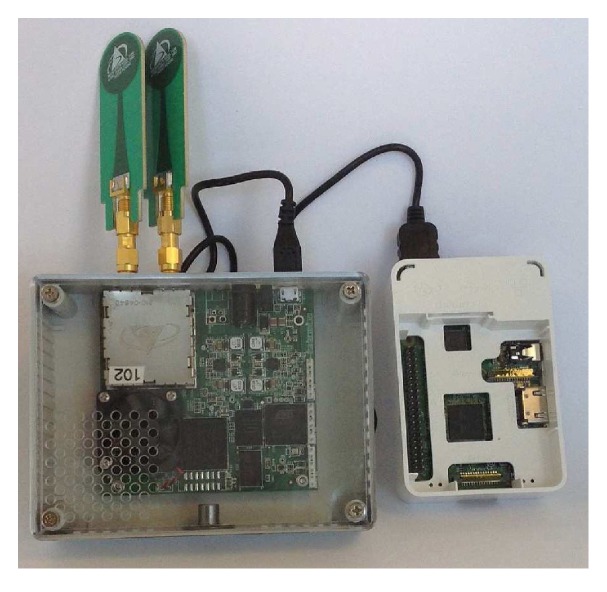
P410 device and associated peripheral hardware.

A Time Domains PulsON 410 (P410) UWB hardware module (shown in Figure 3) is used for the data acquisition purposes. It is a short-range radar with 1.4 GHz of Radio Frequency (RF) bandwidth. This P410 commercial radar module, embedded with in-house developed software was connected to a Raspberry-Pi (RPi) for storing the time stamped radar data. The data have been analyzed and classified offline to compare with ground truth information and correlate the findings. The module transmits at an RF centred frequency of 4.3 GHz with a bandwidth of 2.2 GHz, which follows the Federal Communications Commission (FCC) restrictions [40]. The parameters considered for this experiment are included in Table 1. The pulse integration index (PII) is configured to 12, which is able to integrate $2^{12}=4096$ pulses for a symbol and can provide improved signal to noise ratio (SNR). This device produces base-band pulses of very short duration [41] and transmits pulses at very safe RF levels (−44 dBm/MHz). With appropriate design and signal processing, it can additionally behave as a biosensor and has the added wireless advantage of being able to penetrate through different materials or obstacles so has multiple room effectiveness. In our case, the finite impulse response (FIR) filter is used for the device settings. A 4-tap difference FIR filter has been implemented by convolution for each pulsed wave on each bin where the device takes the first 100 pulsed waves to adjust the filter coefficients and accommodate the background noise. Thus, a 100 data point moving box has been determined by taking each data point from the waves and calculating their average and standard deviation. A detection has been reported by the device when it finds new data with greater average and standard deviation. It has the TW-TOF ranging mechanism that provides precise position information within the short communication range. The single monopole antenna of the radar device set up employs 65 ns TW-TOF which provides an 8 m path radius in all directions. The first 5 ns of the waveform contains jitter because of the direct path interference between the transmitter and receiver antennas. The scan interval is set to 25,000 μs and scans are requested after each interval. The device has a sampling frequency of 16.39 GHz, and a pulse repetition interval (PRI) of approximately 100 ns. The radar performs a scan after each scan interval, which is a function of integration rate and size of scan window. The experiment is carried out using Matlab R2017a tool on an Intel${}^{R}$ Core${}^{\mathrm{TM}}$ i7 processor @ 3.60 GHz running Windows 7 Enterprise 64-bit operating system with a 7856 MB NVIDIA graphics processing unit (GPU).

## 4. Result Analysis

**Figure 4:**
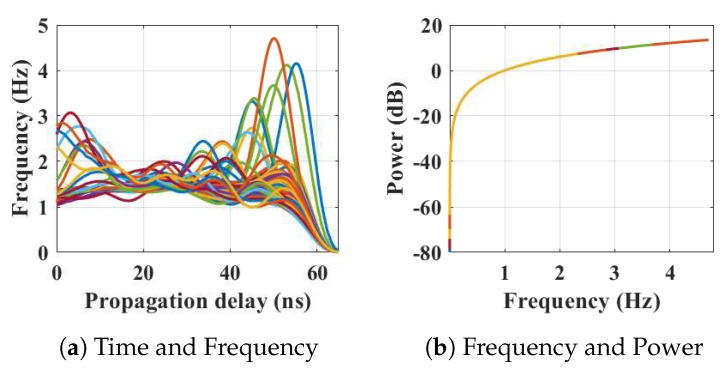
The relationship between propagation delay, activity frequency, and received power from the radar responses obtained while the person is present in the kitchen space has been considered as C1 in classification phase.

**Figure 5:**
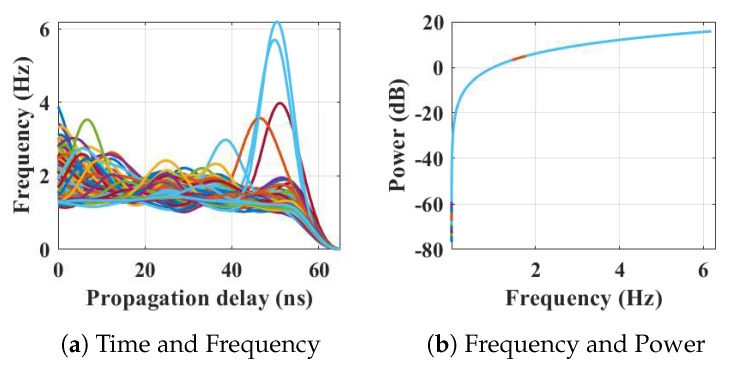
The relationship between propagation delay, activity frequency, and received power from the radar responses obtained while the person is plumping a cushion has been considered as C2 in classification phase.

**Figure 6:**
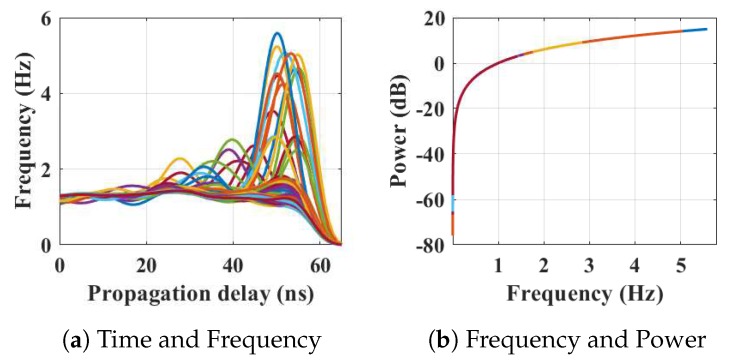
The relationship between propagation delay, activity frequency, and received power from the radar responses obtained while the person is using the microwave in the kitchen has been considered as C3 in classification phase.

**Figure 7:**
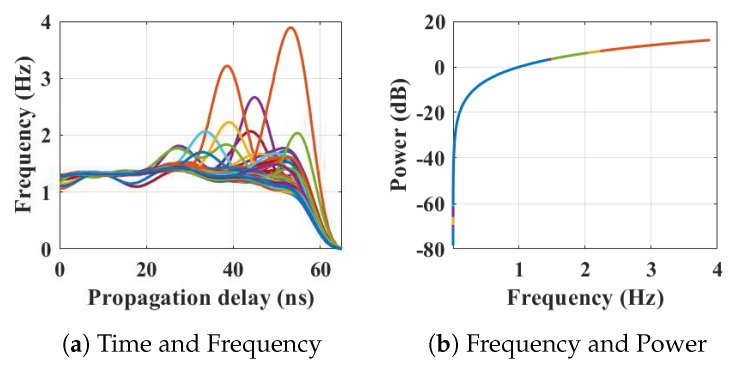
The relationship between propagation delay, activity frequency, and received power from the radar responses obtained while the person is eating in the dining room has been considered as C4 in classification phase.

**Figure 8:**
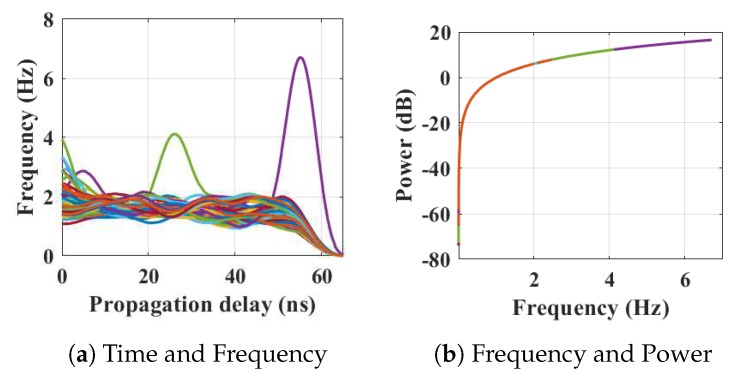
The relationship between propagation delay, activity frequency, and received power from the radar responses obtained while the person is washing a bowl in the kitchen has been considered as C5 in classification phase.

**Figure 9:**
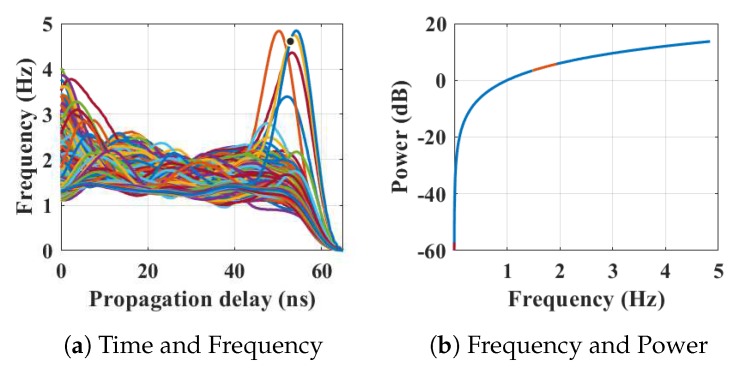
The relationship between propagation delay, activity frequency, and received power from the radar responses obtained while the person is watching television in the living room has been considered as C6 in classification phase.

**Figure 10:**
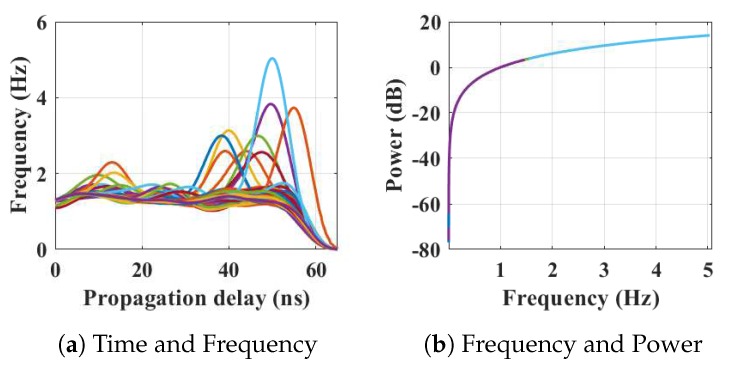
The relationship between propagation delay, activity frequency, and received power from the radar responses obtained while the person is walking from the kitchen through to the dining room and hallway entrance to living room has been considered as C7 in classification phase.

**Figure 11:**
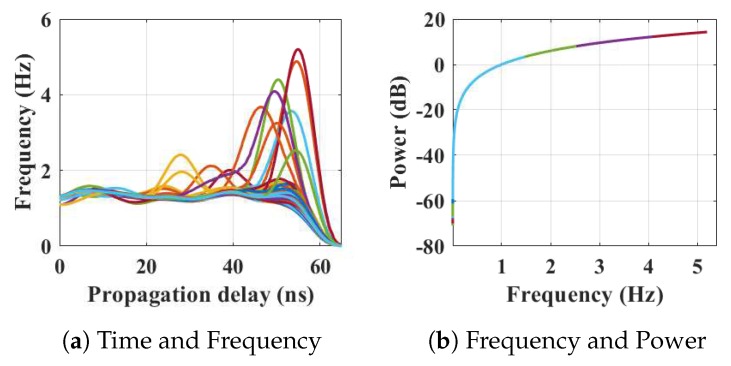
The relationship between propagation delay, activity frequency, and received power from the radar responses obtained while the person is brushing their teeth in the bathroom has been considered as C8 in classification phase.

**Figure 12:**
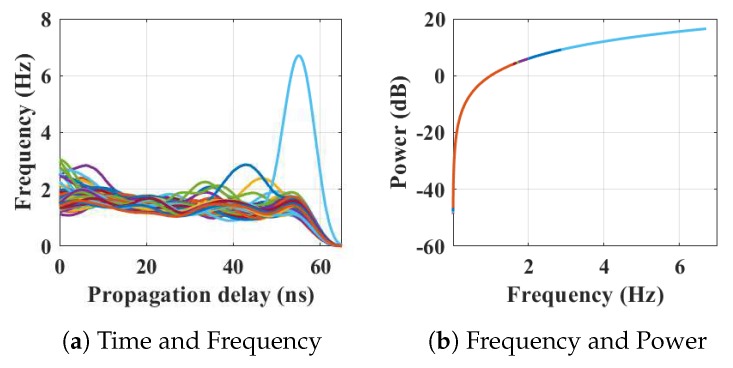
The relationship between propagation delay, activity frequency, and received power from the radar responses obtained while the person is returning from the bathroom to the living room has been considered as C9 in classification phase.

**Figure 13:**
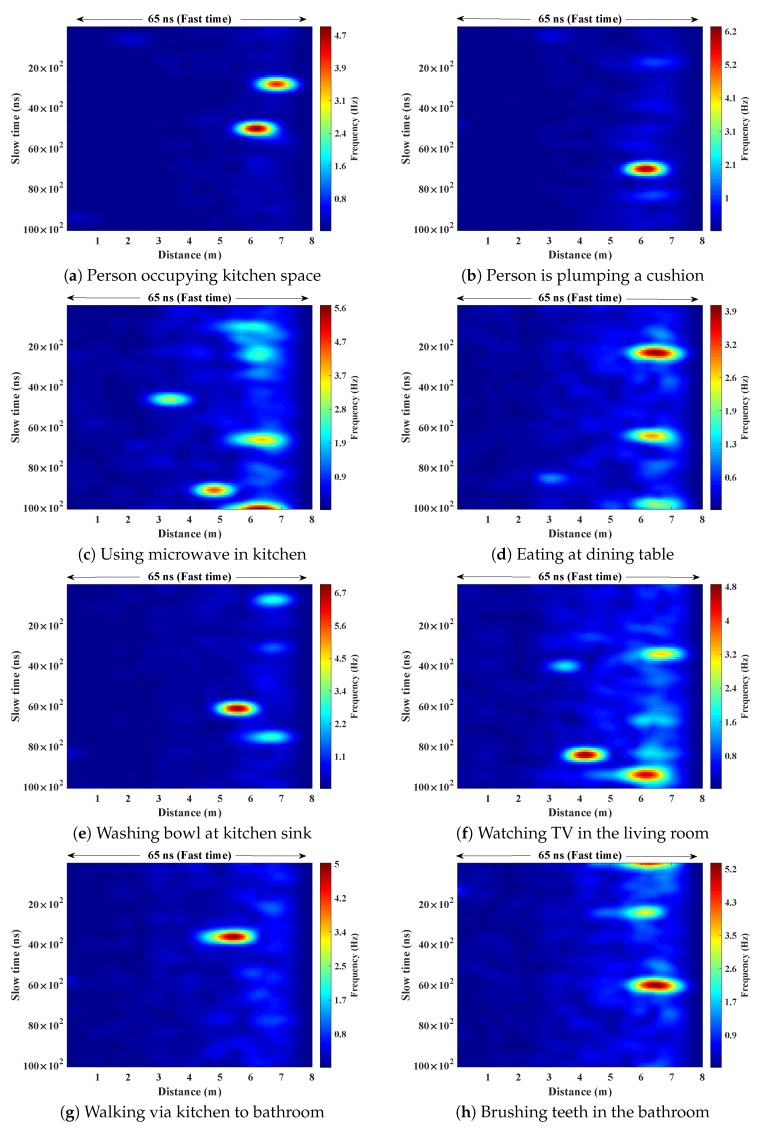

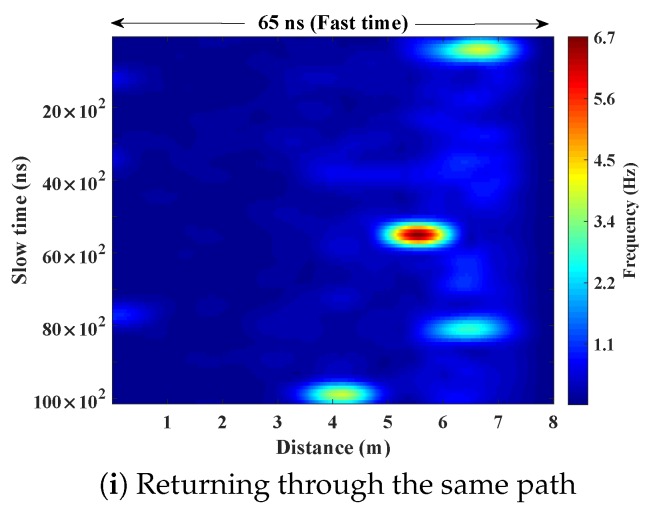
Distance and frequency mapping to agree the floor plan for different categorical events.

Within the home environment under test, nine distinct activities have been considered to identify locations and frequency. This experiment was carried out without local information, but a diary and webcam were used to align outputs post processing to confirm the UWB radar and MC-SVM experimental findings. A single day is considered here to carry out the experiment. There are nine types of radar events processed to represent typical daily household works to be considered for this offline classification task. These nine types of radar events are transformed through STFT to determine the frequency and phase content of scan local sections which varies over time. Figure 4, Figure 5, Figure 6, Figure 7, Figure 8, Figure 9, Figure 10, Figure 11 and Figure 12 describe these events in terms of propagation delay or fast time and frequency over the local sections of a pulsed wave. The propagation delay, or fast time in the current settings, is 65 ns where first 5 ns contain jitter, thus the pulse can travel $\frac{(2.99\times 10^{8}\;m/s)\times 60\;\mathrm{ns}}{2}=8.97$ m with the 60 ns delay. Practically, the radar covers 8 m with this fast time. Moreover, the distance calculation from the micro Doppler signature for each case is shown in Figure 13a–i for better understanding of the scenarios. For each situation, 100 received scans are plotted and color is mapped for visualization, where the highest activity levels have the strongest reds (plumping cushions is red color, sitting still and watching TV are blue color). The slow time or PRI (stated in Table 1) between two pulse is approximately 100 ns, thus total $100\times 10^{2}$ ns of slow time have been labelled in *y*-axis and 8 m of distance has been marked in the *x*-axis.

Figure 4 shows the results when the person is occupying the kitchen space. Figure 4a shows the frequency content of the scans with respect to the time of arrival (ToA), where the frequencies reached 4.7 Hz during movements in that space. The actual position of the person is shown in Figure 13a which is approximately 7 m from the radar with an azimuth of $221^{\circ }$ agreeing with the kitchen floor plan. Figure 4b shows the energy spectrum of that situation.

Subsequently, the person entered in living room after leaving the kitchen. Figure 5 represents the results from the entry and movements in the living room. The participant is asked to carry out typical actions such as, sweeping, dusting, etc. Plumping the cushions for example has the highest frequencies of around 6.2 Hz, where other works (dusting) repeatedly have frequencies under 4 Hz, as shown in Figure 5a. The energy spectrum in Figure 5b displays the power approximately equal to 10 dB. The 2D image plot Figure 13b, shows the frequency contents of these scans with a distance map, where the red color area indicates the position of the person approximately 6.5–7 m away from the radar with an azimuth angle of $268^{{}^{\circ}}$.

Subsequently, the participant entered the kitchen again from the living room via the dining room and began to use the microwave oven; indicated in Figure 6. The received frequencies are up to 5.6 Hz in this case. The distance and azimuth angle are determined through the time vs frequency analysis of Figure 6a. The color map shows that the person is moving between 3–7.2 m over that time period with different azimuths when the corresponding energy expenditure is approximately 18 dB (shown in Figure 6b).

After finishing in the kitchen, the person moved to the dining area to eat at the dining table, where the movements are indicated by peaks in Figure 7a and the corresponding energy spectrum is shown in Figure 7b. It is reflected in the Figure 13d that the movements of the person have the frequency up to 3.9 Hz, but the position and azimuths are approximately the same when the time has changed.

After finishing eating, the person went to kitchen for washing up ppliances. The transformation of scans is shown in Figure 8. Figure 8a represents the time and frequency analysis of the waveforms when the person is washing at the sink. The distance between the person and the radar is roughly 6 m at that time (shown in Figure 13e) with an angle of $225^{{}^{\circ}}$ from the north face of the radar. The corresponding energy is displayed Figure 8b is 19 dB. Further work is ongoing to identify the actual signature of washing up and eating and this would have a dramatic impact on the area of assistive living and monitoring.

Following that, the person moved to the living room from the kitchen and started watching television while sitting on the sofa. The radar events are specified in Figure 9. Here, the frequency responses of below 0–2.5 Hz due to lack of movement at the time of watching television. Sudden movements (e.g., retrieving the remote control) occur during that testing time results frequency contents between 2.5–5 Hz (shown in Figure 9a) are also observed. Figure 9a shows the position of the person is between 3.5–7 m.

After a while, the person left the living room and moved through the hallway entrance towards the bathroom. The transformation of scans and their peaks of the Figure 10a indicates the walking frequency of the person around the house at that time with a different azimuth. Figure 13g represents the frequency with respect to distance.

In the next scenario, the person went to the bathroom for brushing teeth. The received scan responses are analyzed and plotted in Figure 11a. The person is roughly 6–7 m (shown in Figure 13h) from the radar with an azimuth angle of 315°.

Finally, the person moved through the corridor from bathroom to the living room, and the radar responses are analyzed to extract the frequency contents for that, shown in Figure 12a. At that time, the distance of the person from the radar is approximately 7 m. Comparatively high frequencies are shown in the frequency and distance plot of Figure 13i.

Each of the raw scans contains 1152 amplitudes. Pre-processed and transformed scans contain frequency variations of the respective actions with 1064 data points assuming no jitter. Physically these frequencies represent different actions within 8 m, which needs 65 ns of TW-TOF, as shown in Figure 4, Figure 5, Figure 6, Figure 7, Figure 8, Figure 9, Figure 10, Figure 11, Figure 12 and Figure 13. Subsequently, these scans have been transformed to create the feature vectors to train the chosen supervised machine learning (ML) method. The nine categories which have been considered for supervised ML are included in Table 2. Each of these events have been represented by range, azimuth, and frequency of the action and considered the combination as features for the ML phase. Three hundred and fifty-five frequency data points have been extracted after STFT and determined range and azimuth for each of these frequencies. The feature vectors aim to represent an event with its frequency, distance (range) from the radar, and AoA of the pulses. Thus, 355×3=1065 lengths of feature vector have been formed from each radar pulsed scan to describe an event. Thus, the final feature vector from each scan has been considered as $f_{1}\cdots f_{1065}$.

**Figure 14:**
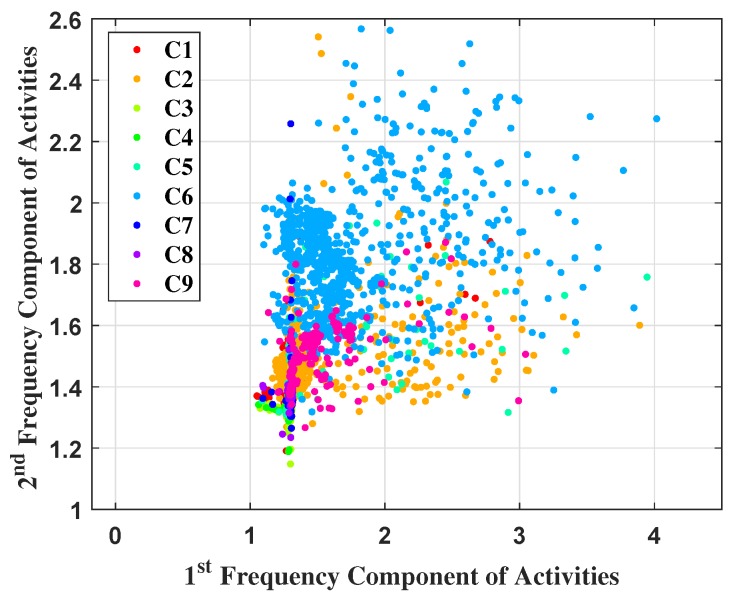
Scatter plot of categorical UWB localization data.

The data prior to ML technique selection have been visualized in Figure 14. It demonstrates a two dimensional representation of the feature vector, where only first two features ($f_{1}$ and $f_{2}$) have been plotted. Physically these two features demonstrate frequency variation of an activity at distances of 0.0091 m and 0.0183 m. The *x* and *y* axis of Figure 14 have been labelled for better understanding. It has been found that the data is distributed in a way which cannot be classified by any linear functioned ML. Therefore, the MC-SVM with quadratic kernel has been chosen, which provides a non-linear decision boundary for classification which has been found to work very well for the situation under investigation. The data have been classified by MC-SVM and outcomes illustrate its capability to predict the locations. The categories C1 to C9 have been described earlier (in Section 4. The 2D plot shows the feature values are very close to each other for some cases, although they belong to different classes.

This imbalanced data distribution makes the classification task difficult for some categories, which is reflected in the confusion matrix later. The data have been randomly partitioned into the training and testing sets. The amount of training data has been altered from 10% to 40%, when testing data amount is 90% to 60%. Each time, the algorithm has been trained by these percentages, tested, and validated by the remaining data. The prediction results have been validated by statistical metrics and entered in Table 3. The averages are taken for each metric and listed here. It shows that the proposed predictive model provided the highest testing correction rate of 0.9047 (marked in bold) and lowest error rate of 0.0953 for the 30% percent training data level.

The testing correction rate increased from 0.8932 to 0.9047 for 10% to 30% training data. The amount of training data was increased with the expectation that accuracy would increase. However, with the 40% training data, the algorithm has over-fitted due to the high dimensionality of the feature vectors, resulting in the testing accuracy decreasing to 0.8963 and the error rate increasing to 0.1037. The objective of the proposed method is to fit the model with the dataset so that it could make valid predictions on new data. Therefore, the performance of the proposed algorithm at 30% training data is considered as the optimal performance of the model. Other evaluation parameters are also determined to support the robustness of the model. In this case (30% training and 70% testing data), sensitivity 0.9038 of the proposed model indicates the probability of correctly identifying the location of the person. Additionally, specificity of 0.9941 tells the probability of the system to recognize the scenario accurately when there are no activities happen in a room. The positive predictive value (PPV) of 0.9695 signifies the probability that the system gives positive results regarding a person’s location from a specific activity, and the true occupancy of the person, and also the negative predictive value (NPV) of 0.9805 points out the probability that system gives a negative result (not in the room) about the person’s location and it is true.

**Figure 15:**
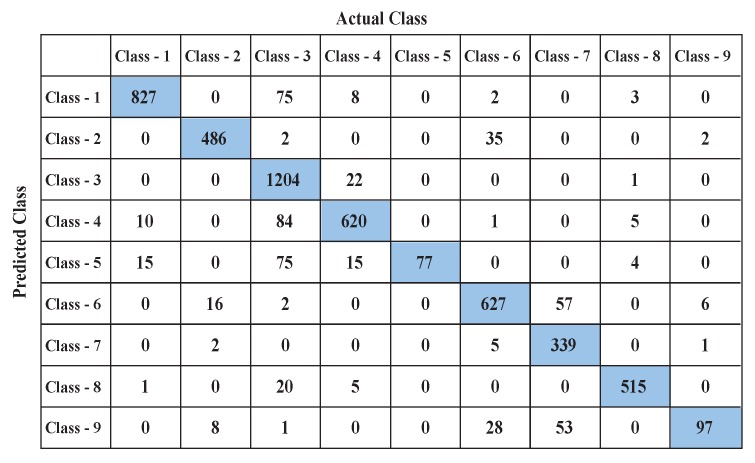
Confusion matrix.

Confusion matrices are observed for further analysis. Figure 15 shows the confusion matrix for learning outcomes when the highest accuracy is achieved (with 30% training and 70% testing data). The classifier has performed very well in case of Classes-1, 2, 3, 6, 7, and 8. These classes (defined previously) actually represent typical activities in a home environment. These data have been gathered from the places where signal attenuation was lower and well within the 8 m radius. Therefore, the locations have been successfully predicted by the MC-SVM for these cases. In addition, the number of false predictions is very low for these categories. The results reveal that most of the misclassification occurred in case of Classes 4, 5, and 9. Dining and kitchen area related signatures are considered as Classes 4 and 5. These two locations are furthest from the single device, beyond thick walls, and are physically contained within the one room space, where the radar suffers a low SNR for detection. This explains the (10 + 84 + 1 + 5) = 100 misidentifications that occurred here.

In the case of the kitchen (Class-5), the total number of misclassifications are (15 + 75 + 15 + 4) = 109, because of the lower SNR and potential multipath confusion that has occurred here. The classifier also became confused in case of Class-9, which considers the walking signature from the bathroom to the living room via the kitchen, dining, and hallway entrances, with (8 + 1 + 28 + 53) = 90 incorrect predictions in this case. In some cases, though the azimuths are different, the frequency content of an activity and distances from the radar are the same, which also leads to the incorrect placement prediction. This work is now considering directional antennas to improve the SNR and reach, and also the implementation of more than one radar device to improve signal levels and accuracy within the real home under investigation.

### 4.1. Comparison

The proposed model’s performance has been compared with recent, similarly aiming works in the field in Table 4. Usually, performance analysis is done via accuracy, specificity or precision, and sensitivity or recall. Thus, these three metrics have been used to create informed comparisons. Yao et al. [16], Diraco et al. [26], and Fleury et al. [28] have implemented either accuracy or sensitivity-specificity. Barsocchi et al. [22] have chosen three best performing localization based AAL namely CPS [23], n-Core [24], and RealTrac [25] from EvAAL to assess their performance. Thus, the performance of these three systems have been provided in Table 4. The performance metrics of the proposed work have been marked in bold font. Barsocchi et al. (CPS) [22,23] has achieved best accuracy of 0.9120 (≡91.20%), while Yao et al. [16] achieved lowest accuracy of 0.7843 (≡78.83%) among other methods listed here. Yao et al. [16] have also performed the work for more than one subject at a time and achieved better accuracy, but the works compared here focused to help or provide assistance per person, thus the performance to assist a single person has been considered from the model of Yao et al. [16]. Though accuracy, specificity, and sensitivity are popular and established metrics, accuracy cannot uniquely quantify a model’s performance because of its consideration of all predictions (including true positive and true negative). Thus, the high accuracy sometimes misleads the performance analysis which is reflected in case of Barsocchi et al. (CPS) [22,23]. The method has not achieved high precision and recall indicating the low positive predictions (true positives) and low positive predictions among each class. The proposed work has attained highest specificity of 0.9941 (≡99.41%) and sensitivity of 0.9038 (≡90.38%), signifying the preciseness and completeness of the proposed model. The models Barsocchi et al. (CPS) [22,23], Barsocchi et al. (n-Core) [22,24], and Barsocchi et al. (RealTrac) [22,25] have attained high accuracy but low specificity and sensitivity indicating an imbalance in performance for different scenarios. Chernbumroong et al. [27] have reached to steady performance in terms of all three metrics. In other references, Lopez-de-Teruel et al. [19], Diraco et al. [26], and Fleury et al. [28] have resulted either a high accuracy or low specificity-sensitivity or vice versa, whereas the proposed work has attained a stable performance in terms of all three metrics and can be therefore be considered as a trusted, well-performing, intelligent AAL model.

### 4.2. Discussion

The proposed ECS prototype intends to observe and track the daily living as well as the working environment to provide safe, active, and independent life for those involved. Usually, the context aware models are restricted for two reasons and require significant advancement; requirements of infrastructure and unwillingness to accept assistive systems. The proposed work has been built by considering these two reasons. The proposed model has used a single non-intrusive IR-UWB biosensor device for monitoring purpose, whereas the existing works need to employ wearable device for each person or time-of-flight cameras, which cannot work in non-line of sight condition, as well be seen to invade user privacy and security. The device has a resolution of 9.15 mm, thus two movements separated by 9.15 mm can be identified in the floor plane with the help of the range and derived azimuth measurement. Therefore, the identification of an exact location of movement is possible, whereas the existing works using RSSI, accelerometers, or wearable device to understand the location. This whole architecture is connected with a secure cloud server mechanism to understand home condition remotely, where the SVM algorithm has been trained to discover different type of movements for household activities. It has attained better performance (accuracy ≡90.47%, specificity ≡99.41%, and sensitivity ≡90.38%) than other state-of-art works to understand and notify in home conditions. Subsequently, no movement for a given time, twitching, jerking, body shaking unusually would provide new patterns to the system could generate notifications for the attention of caregivers. The IR-UWB device has PRI (lsited in Table 1) of 100 ns which means each pulse will repeat after 100 ns, the scan interval (lsited in Table 1) of 25,000 μs, the range is being updated after every 132 ms (in the current settings, PII = 12), and the system takes 3.2 ms to process each scan. Therefore, it takes 160.20 ms to reflect some movement or no-movement in the model. Thus, any decision regarding abnormal occurrences can be taken within this interval. The radar has been fixed to position therefore, it does not need to be carried or considered after deployment which would be easy to accept the system and obtain true behaviour marking for the user since they can effectively forget they are being monitored. Therefore, the ECS model would be a trusted, well performing, and intelligent solution for home monitoring.

## 5. Conclusions and Future Work

An intelligent ECS system employing a UWB radar module with single transmitter and receiver augmented by machine learning approach has been proposed in the context of AAL. This work is theoretically and practically tested. The salient feature of the research is to recognize the locations of an elder person in home from the daily activities. This concept could be employed in AAL applications to improve wellbeing and self-reliance, with non-intrusive assistance embedded to identify falls, changes in daily behavior, etc. that could pinpoint problems early on such a loneliness, expression, dementia, and inactivity.

Users can be tracked remotely using their UWB micro-Doppler signature in home environment without hampering their privacy and comfort. For this purpose, the proposed model has to be trained by the common daily actions with their time stamps. Apart from this, the presented work has some limitations: (i) Only one UWB radar device is considered for the data collection in this case. Hence, beyond 10 m of coverage, signal strength at some positions in the furthest rooms, the kitchen and dining become low and results in misclassification or misidentification of some positions. Additional devices can be used, and the same location awareness system be employed or directional antennas will be investigated for improved SNR. (ii) Detection of low frequencies is difficult when employing STFT, whereas short pulses are difficult to be localized in time with long windows. In addition, the fixed size window length for convolution is not always appropriate. These restrictions will be overcome in the future work. Improvements in resolution and ranging information for each room will be investigated, also the week and month data will be considered to improve long-term performance. The continuous wavelet transform (CWT) will also be examined for better time-frequency analysis.

## Figures and Tables

**Table 1:** Parameter setting for the monostatic ultra wide band (UWB) radar module.

Parameter	Values
Center frequency	4.3 GHz
Frequency range	3.1 GHz to 5.3 GHz
PII	12
Sampling frequency	16.39 GHz
PRI	approximately 100 ns
Scan time interval	25,000 μs
Transmit gain	−12.64 dBm
Radar area coverage	upto 10 m
Number of antennas	2 [$T_{x}$ and $R_{x}$]

**Table 2:** The categories, description, and the features used for event classification by support vector machine (SVM).

Class Name	Class Description	Feature Description
C1	The person is moving in the kitchen area.	The feature vectors have been made by concatenating range, azimuth, and corresponding frequency obtained from STFT. Therefore, 1065 features have been concatenated for one feature vector where, 355 features have been derived to represent each frequency, range, and azimuth.
C2	The person is plumping cushions in the living room.	
C3	The person is using the microwave in the kitchen.	
C4	The person is eating at the dining table.	
C5	The person is washing up at the kitchen sink.	
C6	The person is watching television in the living room.	
C7	The person is walking from the kitchen to the bathroom via dining room, entrance, and living room.	
C8	The person is brushing teeth in the bathroom.	
C9	The person is returning via the same path described in C7.	

**Table 3:** Classification result of the proposed method.

Statistical Measurements	10%	20%	30%	40%
Correct Rate	0.8932	0.8946	**0.9047**	0.8963
Error Rate	0.1068	0.1054	0.0953	0.1037
Sensitivity	0.8995	0.9037	0.9038	0.9010
Specificity	0.9949	0.9951	0.9941	0.9948
Positive Predictive Value	0.9721	0.9735	0.9695	0.9705
Negative Predictive Value	0.9803	0.9812	0.9805	0.9815
Area Under the Curve	0.6087	0.6183	0.6245	0.6195
Time elapsed (in Seconds)	3.6148	3.1795	3.0573	2.5555

**Table 4:** Comparison of outcomes with other state-of-art methods.

Methods	Accuracy	Specificity	Sensitivity
Yao et al. [16]	0.7843	-	-
Lopez-de-Teruel et al. [19]	0.9000	0.9300	0.8000
Barsocchi et al. - CPS [22,23]	0.9120	0.6860	0.7770
Barsocchi et al.- n-Core [22,24]	0.9060	0.6600	0.7950
Barsocchi et al.- RealTrac [22,25]	0.8950	0.6230	0.7950
Diraco et al. [26]	-	0.8015	0.8727
Chernbumroong et al. [27]	0.9023	0.9043	0.9022
Fleury et al. [28]	0.8620	-	-
Proposed prototype	**0.9047**	**0.9941**	**0.9038**

## References

[1] Kleinberger T., Becker M., Ras E., Holzinger A., Muller P. (2007). Ambient intelligence in assisted living: Enable elderly people to handle future interfaces. International Conference on Universal Access in Human-Computer Interaction.

[2] Erden F., Velipasalar S., Alkar A.Z., Cetin A.E. (2016). Sensors in Assisted Living: A survey of signal and image processing methods. IEEE Signal Process. Mag..

[3] Patwari N., Hero A.O., Perkins M., Correal N.S., O’dea R.J. (2003). Relative location estimation in wireless sensor networks. IEEE Trans. Signal Process..

[4] Rana S.P., Prieto J., Dey M., Dudley S.E.M., Rodríguez J.M.C. (2018). A Self Regulating and Crowdsourced Indoor Positioning System through Wi-Fi Fingerprinting for Multi Storey Building. Sensors.

[5] Ali A.M., Asgari S., Collier T.C., Allen M., Girod L., Hudson R.E., Yao K., Taylor C.E., Blumstein D.T. (2009). An empirical study of collaborative acoustic source localization. J. Signal Process. Syst..

[6] Martino L., Míguez J. (2010). Generalized rejection sampling schemes and applications in signal processing. Signal Process..

[7] Jokanovic B., Amin M.G., Zhang Y.D., Ahmad F. (2014). Multi-window time–frequency signature reconstruction from undersampled continuous-wave radar measurements for fall detection. IET Radar Sonar Navig..

[8] Ozcan K., Mahabalagiri A.K., Casares M., Velipasalar S. (2013). Automatic fall detection and activity classification by a wearable embedded smart camera. IEEE J. Emerg. Sel. Top. Circuits Syst..

[9] Silva B.M., Rodrigues J.J., Simoes T.M., Sendra S., Lloret J. An ambient assisted living framework for mobile environments. Proceedings of the 2014 IEEE-EMBS International Conference on Biomedical and Health Informatics (BHI).

[10] Zhou Z., Chen X., Chung Y.C., He Z., Han T.X., Keller J.M. (2008). Activity analysis, summarization, and visualization for indoor human activity monitoring. Comput. Electr. Eng. Publ..

[11] Mrazovac B., Bjelica M.Z., Papp I., Teslic N. Smart audio/video playback control based on presence detection and user localization in home environment. Proceedings of the 2011 2nd Eastern European Regional Conference on the Engineering of Computer Based Systems (ECBS-EERC).

[12] Bourke A.K., Prescher S., Koehler F., Cionca V., Tavares C., Gomis S., Garcia V., Nelson J. (2012). Embedded fall and activity monitoring for a wearable ambient assisted living solution for older adults. Conf. Proc. IEEE Eng. Med. Biol. Soc..

[13] Uenoyama M., Matsui T., Yamada K., Suzuki S., Takase B., Suzuki S., Ishihara M., Kawakami M. (2006). Non-contact respiratory monitoring system using a ceiling-attached microwave antenna. Med. Biol. Eng. Comput..

[14] Tsirmpas C., Anastasiou A., Bountris P., Koutsouris D. (2015). A new method for profile generation in an internet of things environment: an application in ambient-assisted living. IEEE Internet Things J..

[15] Costa S.E., Rodrigues J.J., Silva B.M., Isento J.N., Corchado J.M. (2015). Integration of wearable solutions in aal environments with mobility support. J. Med. Syst..

[16] Yao B., Hagras H., Alghazzawi D., Alhaddad M.J. (2016). A big bang-big crunch type-2 fuzzy logic system for machine-vision-based event detection and summarization in real-world ambient-assisted living. IEEE Trans. Fuzzy Syst..

[17] Diamantini C., Freddi A., Longhi S., Potena D., Storti E. (2016). A goal-oriented, ontology-based methodology to support the design of AAL environments. Expert Syst. Appl..

[18] Alcalá J.M., Ureña J., Hernández Á., Gualda D. (2017). Sustainable Homecare Monitoring System by Sensing Electricity Data. IEEE Sens. J..

[19] Lopez-de Teruel P.E., Garcia F.J., Canovas O., Gonzalez R., Carrasco J.A. (2017). Human behavior monitoring using a passive indoor positioning system: a case study in a SME. Procedia Comput. Sci..

[20] Bleda A.L., Fernández-Luque F.J., Rosa A., Zapata J., Maestre R. (2017). Smart sensory furniture based on WSN for ambient assisted living. IEEE Sens. J..

[21] Hassan M.K., El Desouky A.I., Elghamrawy S.M., Sarhan A.M. (2018). Intelligent hybrid remote patient-monitoring model with cloud-based framework for knowledge discovery. Comput. Electr. Eng..

[22] Barsocchi P., Cimino M.G., Ferro E., Lazzeri A., Palumbo F., Vaglini G. (2015). Monitoring elderly behavior via indoor position-based stigmergy. Pervasive Mob. Comput..

[23] Bocca M., Kaltiokallio O., Patwari N. (2012). Radio tomographic imaging for ambient assisted living. International Competition on Evaluating AAL Systems through Competitive Benchmarking.

[24] Tapia D.I., García Ó., Alonso R.S., Guevara F., Catalina J., Bravo R.A., Corchado J.M. (2011). The n-core polaris real-time locating system at the evaal competition. International Competition on Evaluating AAL Systems through Competitive Benchmarking.

[25] Moschevikin A., Galov A., Soloviev A., Mikov A., Volkov A., Reginya S. (2013). Realtrac technology overview. International Competition on Evaluating AAL Systems through Competitive Benchmarking.

[26] Diraco G., Leone A., Siciliano P. (2017). A radar-based smart sensor for unobtrusive elderly monitoring in ambient assisted living applications. Biosensors.

[27] Chernbumroong S., Cang S., Atkins A., Yu H. (2013). Elderly activities recognition and classification for applications in assisted living. Expert Syst. Appl..

[28] Fleury A., Vacher M., Noury N. (2010). SVM-based multimodal classification of activities of daily living in health smart homes: sensors, algorithms, and first experimental results. IEEE Trans. Inf. Technol. Biomed..

[29] Rana S.P., Dey M., Siddiqui H.U., Tiberi G., Ghavami M., Dudley S. UWB Localization Employing Supervised Learning Method. Proceedings of the 17th IEEE International Conference on Ubiquitous Wireless Broadband ICUWB.

[30] Rana S.P., Dey M., Brown R., Siddiqui H.U., Dudley S. Remote vital sign recognition through machine learning augmented UWB. Proceedings of the European Conference on Antennas and Propagation, Excel London, Docklands.

[31] Saeed A., Kosba A.E., Youssef M. (2014). Ichnaea: A low-overhead robust WLAN device-free passive localization system. IEEE J. Sel. Top. Signal Process..

[32] Zhong J., Huang Y. (2010). Time-frequency representation based on an adaptive short-time Fourier transform. IEEE Trans. Signal Process..

[33] Nawab S.H., Quatieri T.F. (1987). Short-time Fourier transform. Advanced Topics in Signal Processing.

[34] Richards M.A. (2005). Fundamentals of Radar Signal Processing.

[35] Crammer K., Singer Y. (2001). On the algorithmic implementation of multiclass kernel-based vector machines. J. Mach. Learn. Res..

[36] Dey M., Rana S.P., Dudley S. (2018). Smart building creation in large scale HVAC environments through automated fault detection and diagnosis. Future Gen. Comput. Syst..

[37] Powers D.M. (2011). Evaluation: From Precision, Recall and F-Measure to ROC, Informedness, Markedness and Correlation. http://hdl.handle.net/2328/27165.

[38] Brown R., Ghavami N., Siddiqui H.U.R., Adjrad M., Ghavami M., Dudley S. (2017). Occupancy based household energy disaggregation using ultra wideband radar and electrical signature profiles. Energy Build..

[39] Vastardis N., Kampouridis M., Yang K. (2016). A user behaviour-driven smart-home gateway for energy management. J. Ambient Intell. Smart Environ..

[40] Federal Communications Commission (2002). In the Matter of Revision of Part 15 of the Commission’s Rules Regarding Ultra-Wideband Transmission Systems. https://www.gpo.gov/fdsys/pkg/FR-2010-10-12/xml/FR-2010-10-12.xml.

[41] Win M.Z., Scholtz R.A. (1998). Impulse radio: How it works. IEEE Commun. Lett..

